# Mobile ultra-clean unidirectional airflow screen reduces air contamination in a simulated setting for intra-vitreal injection

**DOI:** 10.1007/s10792-016-0236-1

**Published:** 2016-04-30

**Authors:** Ruth Lapid-Gortzak, Roberto Traversari, Jan Willem van der Linden, Sarit Y. Lesnik Oberstein, Oren Lapid, Reinier O. Schlingemann

**Affiliations:** 1 Department of Ophthalmology, Academic Medical Center, University of Amsterdam, Meibergdreef 9, 1100 AZ Amsterdam, The Netherlands; 2 Retina Total Eye Care, Driebergen, The Netherlands; 3 TNO, Dutch Center for Health Assets, Soesterberg, The Netherlands; 4 Dept of Plastic and Reconstructive Surgery, Academic Medical Center, University of Amsterdam, Amsterdam, The Netherlands

**Keywords:** Unidirectional ultra-clean laminar airflow, Intra-vitreal injections, Endophthalmitis prevention, HEPA filters, Contamination

## Abstract

The aim of this study is to determine whether the use of a mobile ultra-clean laminar airflow screen reduces the air-borne particle counts in the setting of a simulated procedure of an intra-vitreal injection. A mobile ultra-clean unidirectional airflow (UDF) screen was tested in a simulated procedure for intra-vitreal injections in a treatment room without mechanical ventilation. One UDF was passed over the instrument tray and the surgical area. The concentration of particles was measured in the background, over the instrument table, and next to the ocular area. The degree of protection was calculated at the instrument table and at the surgical site. Use of the UDF mobile screen reduced the mean particle concentration (particles > 0.3 microns) on the instrument table by a factor of at least 100.000 (*p* < 0.05), and over the patient’s eye by at least a factor of 436 (*p* < 0.05), which in clinical practice translates into significantly reduced air contamination. Mobile UDF screen reduces the mean particle concentration substantially. The mobile UDF screen may therefore allow for a safer procedural environment for ambulatory care procedures such as intra-vitreal injections in treatment rooms.

## Introduction

Intra-vitreal injections are commonly performed ambulatory treatments. The risk of infection, according to the literature, is between 0.2 and 0.03 %. [1–3] However, the risk is repetitive and cumulative due to the need for repeated injections in this patient population. Notwithstanding the low incidence of infection, this complication is devastating to the patient, and difficult and expensive to treat. Most infections are caused by the patients’ or health workers skin flora. [4, 5] Topical prophylaxis with antibiotic drops has been shown to be ineffective secondary to bacterial resistance [6, 7].

It has been suggested that when performing injections, aseptic techniques need to be adhered to in addition to povidone iodine rinsing and antibiotic eye drops [1]. Nevertheless, international guidelines include variable instructions for the surroundings in which intra-vitreal injections need to be done. The British Royal College of Ophthalmology guidelines mandate that the procedure needs to be done in an operating room, or a dedicated treatment room [1]. The policy statement of the American Academy of Ophthalmology does not set guidelines for the immediate environment in which the intra-vitreal injection should take place [2]. Outside ophthalmology, the importance of air-borne particles in causing infection has been proven in orthopedic implant surgery, where the infection rates have been significantly reduced using measures to decrease air-borne particle concentration [4].

Measures aimed at reducing air contamination include surgical garb for the team [5–7], and proper operating theater ventilation [5]. The purpose of air treatment is to prevent stagnation of air and its contaminants, removal of air-borne contaminants, and to provide a comfortable environment for the patient and surgical team [5]. Over the years, the concept of operating boxes has been developed in which a smaller area of ultra-clean laminar airflow was utilized to reduce infection rates [8]. The use of mobile ultra-clean unidirectional airflow screens (UDF) has added value in settings where the existing ventilation does not suffice and in situations where the mobile screen may be an independent means of achieving clean air in the surgical area, when the procedure is not deemed to necessitate a full operating theater setting [4, 8–10]. This is the situation of intra-vitreal injections.

Air is contaminated by not only particles, mostly skin scales, but also bacteria. In simulation studies of contaminated air as well as in air samples in ultra-clean operating theaters, it has been shown that there is a good correlation between particles of 5–7 microns and the amount of microorganisms present in the air [11]. These particle sizes have the same behavioral characteristics in flowing air as the larger but much rarer bacteria-carrying particles [12]. Counting these 5–7 micron particles, which are present in higher numbers and can be measured more reliably, is therefore considered to provide a good estimate of the presence of larger particles [11]. In addition, in simulated circumstances, it is very difficult to produce enough large particles, and as a result, standardized measurements in simulated conditions are being done on the smaller particles.

The purpose of our study was to evaluate whether the use of a commercially available mobile screen UDF with a unidirectional HEPA 14 filtered laminar air flow will decrease levels of air contamination on the instrument table as well as the surgical area, in a simulated model for intra-vitreal injections.

## Methods

**Fig. 1:**
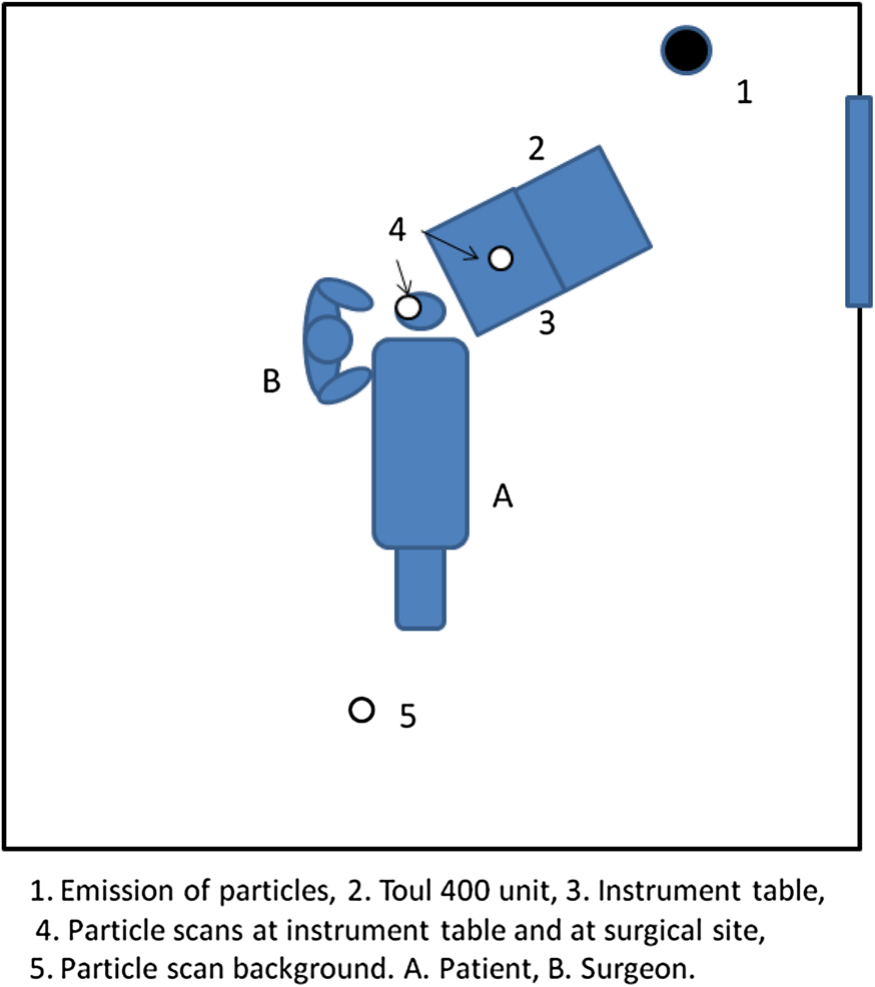
This figure schematically renders the setup of the measurements in the operating theater. The larger *black dot* is the site of particle emission and measurement of background particles, while the smaller *white dots* are the sites where the measurements were done on the instrument table and near the operative site, i.e., the intra-vitreal injection site

A simulation of an intra-vitreal injection was performed in an operating room (OR). The OR was used as an environment for simulation. The door closed, and all s-control systems turned off. As a result, the head position of the patient is not consequential, as the particles, like gas particles, are diffusely and evenly spread in the room. [13, 14] A setup was arranged in which particles were emitted on one side of the OR, and particles were measured at 3 sites: (1) at the instrument table, (2) at the operative site, and (3) at the other side of the patient, representing the background measurement (Fig. 1). In this approach, the particles are spread throughout the space, and the measured concentration at the reference point is lower than that which the system is exposed to [15], as such the effect of emitting at different positions is very limited. The UDF unit was placed in such a manner, that the air flow was directed in sequence from the unit, over the instrument table and draping onto the operative site with no mechanical barriers on its path. The mechanical ventilation system of the operating room was switched off so the only air circulation and filtration was established by the UDF unit. Air-borne particles were measured on the instrument table, at the operative site, and in the background, both at rest with a volunteer and surgeon in place without movement, and with a sham procedure in place, with the surgeon simulating the actual movements, but without actual instruments. The air was sampled at 30-s intervals and simultaneously measured with 3 particle counters (Lighthouse3016-IAQ, Fremont, Ca, USA) for particles in the size categories of ≥0.3 µm, ≥0.5 µm, ≥1.0 µm, ≥2.5 µm, ≥5.0 µm, and ≥10.0 µm. A steady high background flow of particles was obtained by the vaporization of tap water with an ultrasonic fogger (Lighthouse Volcano P6). After vaporization of the water droplets emitted by the machine, the solid particles remain air borne in the air flow in the room. The background concentration (particles/scan) was measured at this site. Outcome parameters are the reduction in the number of particles at the instrument table and at the ocular surface, and the reduction in these as compared to the background concentration.

**Fig. 2:**
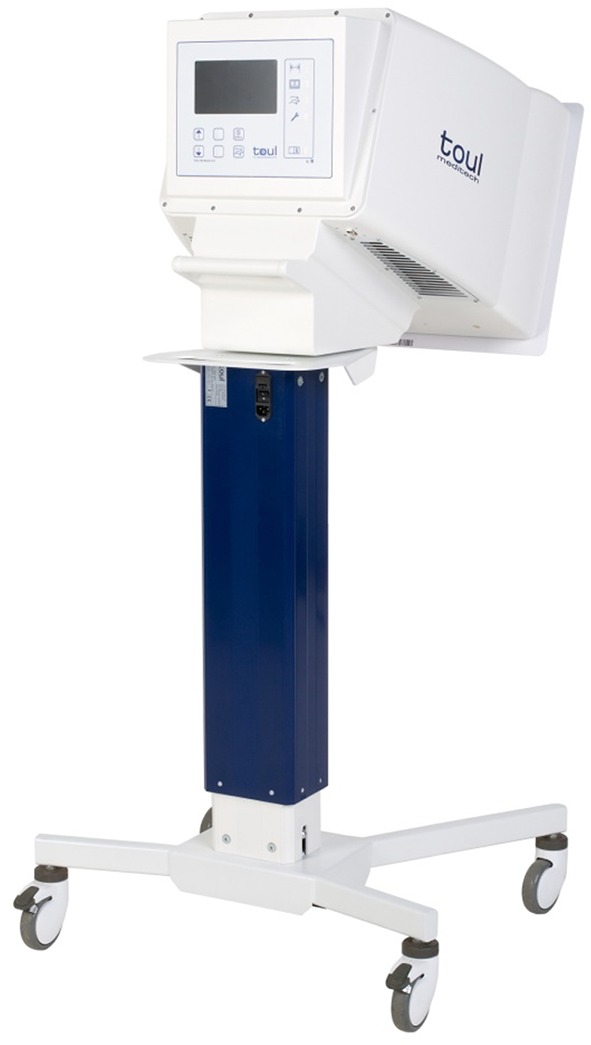
The Toul 400 mobile ultra-clean laminar air flow unit. On top of the standard equipped with 4 wheels is the flow unit with the HEPA filter. Positioning of the flow is done by placing the unidirectional laminar flow device adjacent to the area that needs to be treated

The area at the instrument table and at the surgical site were covered by flow from the unidirectional flow unit, which has an air discharge opening of 37 × 49.5 cm, airflow of 400 m^3^/h, and producing an airspeed of 0.5–0.7 m/s (Fig. 2). The HEPA H14 filter was used, with 99.995 % efficiency for filtering the most penetrating particles from the air [ISO 29463, High-efficiency filters and filter media for removing particles in air].

The protective factor of this system was derived according to the following formula [16]:

$${\text{DP}}_{x} = \, - \log \, \left( {C_{x} /C_{\text{ref}} } \right),$$

where DP_*x*_ is the degree of protection at position *x, C*_*x*_ is the concentration of particles in the “clean” area at position *x*, and *C*_ref_ is the concentration of particles outside the “clean” area, i.e., the background.

A degree of protection at location of 0 means that the particle concentration in the operative field is the same as in the background. A protective factor of 4 means that there is a reduction of a factor 10.000 of particles in the clean area compared to the background area. A negative factor means that the operative area carries more particles than the background area.

The degree of protection in our analysis was limited (truncated) to a factor 5 for the situation in which no particles were found in the location x, the clean area. This means a reduction factor of at least 100.000.

The ISO14644-1 standard shows the correlation between air sampling size, number of background particles measured, and number of particles counted in order to be able to calculate a degree of protection for a minimal sample volume [17]. Under the circumstances measured, in which the sample volume was 28.3 dm^3^ for the amount of particles measured for the size of 0.3 micron particle, a degree of protection of 5 is statistically significant. For particle sizes of 0.5 microns, a degree of protection of 3.5 is statistically significant. For particles of 1 micron, a degree of protection of 2.5 is statistically significant. As stated in the ISO 14644-1, a sufficient volume of air should be sampled in order to be able to detect a minimum of 20 particles, which is defined as the limit for the designated ISO class. Using this method, a back calculation was performed in order to be able to establish the reliability of the degree of protection that was found. During each of the measurements at least 28.8 dm^3^ [cubic foot] of air was sampled.

## Results

The simulation included 10 measurements of the background particle concentration. The measurement of the particle count over the instrument table and surgical site at rest was repeated 4 times, while the same setup with the simulated intervention was measured 10 times.

**Fig. 3:**
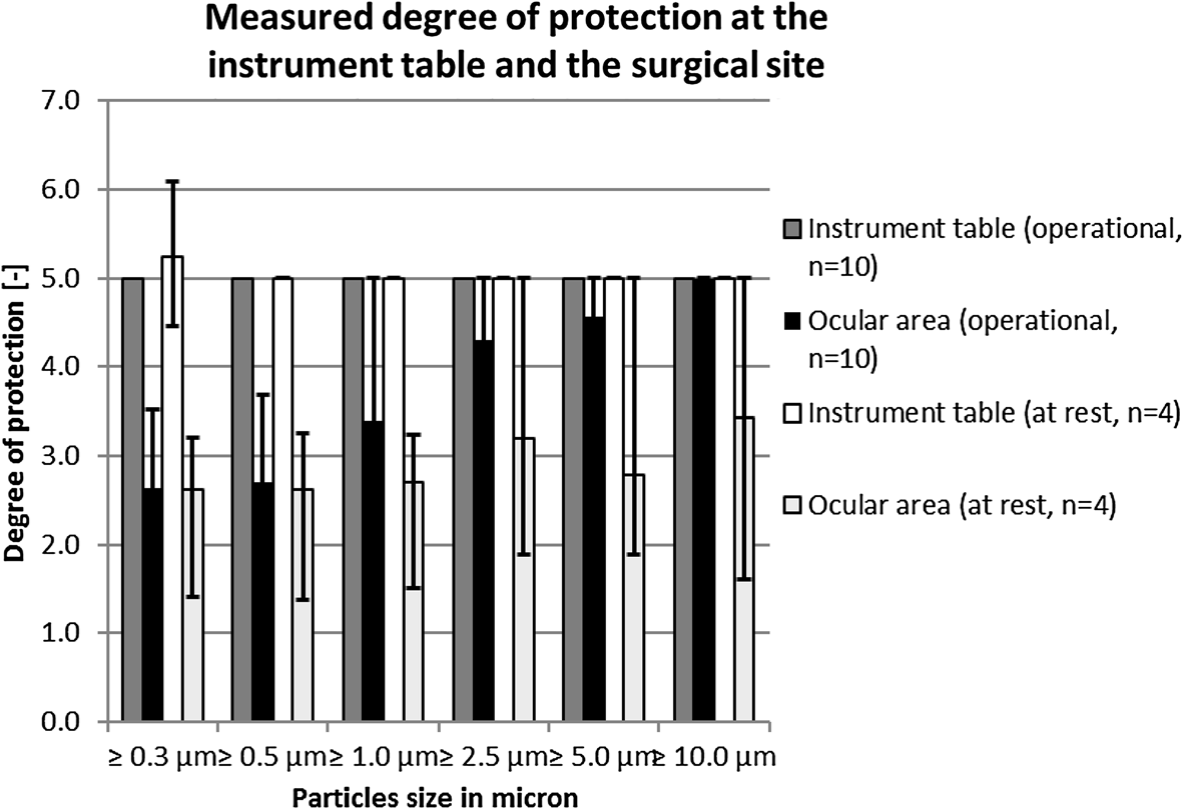
This figure shows the degree of protection at the ocular area and on the instrument table, comparing the working state to the resting state

A significant degree of protection could be demonstrated for the simulated procedure both at the operating table and at the instrument table. At the instrument table, the degree of protection was constant and maximal at a value of 5.00 (Table 1). At the operative area, the degree of protection measured was between 2.64 (CI 95 % 2.31–2.96) for 0.3 micron particles, to a constant value of 5.00 for 10 micron particles. In Fig. 3, the degrees of protection are rendered graphically.

**Table 1:** The measured degree of protection with the UDF mobile and the 95 % confidential interval during the at rest and operational conditions is shown here

Particle size	≥0.3 µm	≥0.5 µm	≥1.0 µm	≥2.5 µm	≥5.0 µm	≥10.0 µm
Instrument table (operational, *n* = 10)						
Mean value	5.00^a,b^	5.00^b^	5.00^b^	5.00^b^	5.00^b^	5.00^b^
Standard deviation						
95 % CI upper boundary						
95 % CI lower boundary						
Instrument table (at rest, *n* = 4)						
Mean value	5.25^**a**^	5.00^b^	5.00^b^	5.00^b^	5.00^b^	5.00^b^
Standard deviation	0.69					
95 % CI upper boundary	6.34					
95 % CI lower boundary	4.15					
Ocular area (operational, *n* = 10)						
Mean value	2.64	2.70	3.39	4.31	4.57^a^	5.00^b^
Standard deviation	0.45	0.50	1.39	1.46	1.37	
95 % CI upper boundary	2.96	3.05	4.38	5.35	5.55	
95 % CI lower boundary	2.31	2.34	2.39	3.27	3.58	
Ocular area (at rest, *n* = 4)						
Mean value	2.63	2.63	2.70	3.19	2.78^a^	3.43
Standard deviation	0.83	0.84	0.82	1.34	1.49	1.83
95 % CI upper boundary	3.95	3.97	4.00	5.32	5.15	6.33
95 % CI lower boundary	1.31	1.28	1.39	1.07	0.41	0.52

*CI* confidence interval

^a^Values differ significantly (*P* < .05)

^b^Constant truncated value (no particles ware detected)

Table 1 shows that the degree of protection at rest (when measurements were done without movement of the surgeon over the instrument tray or the surgical site) was lower than that in the simulated injection procedure. However, the influence of the simulated procedure was small and the difference in the degree of protection was only significant (*p* < 0.05) for the ≥0.3 micrometer particles on the instrument table and the ≥5.0 micrometer particles at the ocular area. For the ≥5.0 micrometer particles, the background concentration between the different situations differed significantly (*p* < 0.05) too, but because of the low incidence of the larger particles in the background, it is more difficult to get a significant reduction in their already low numbers.

**Fig. 4:**
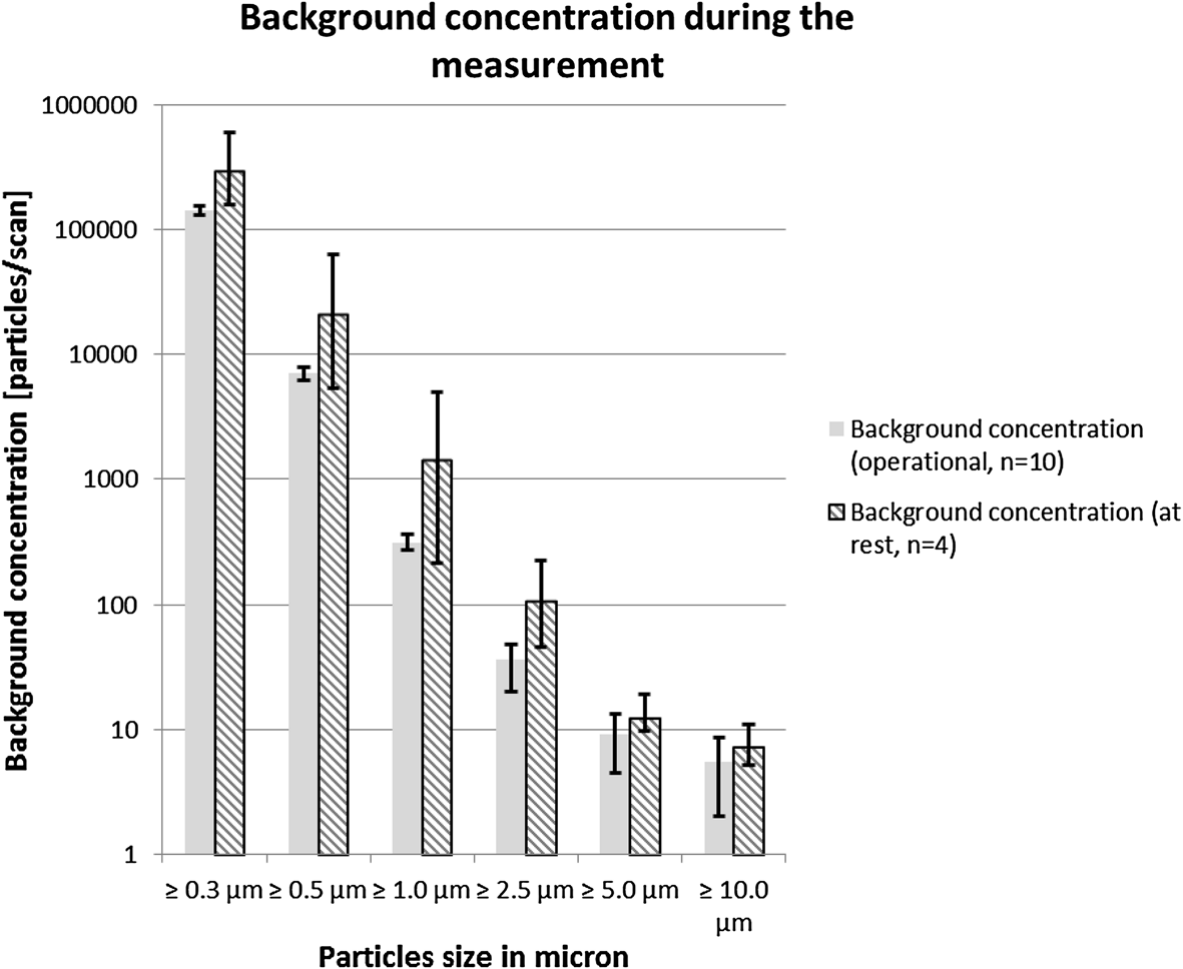
This figure shows the background concentration of particles both during the sham procedure and at rest. The smallest particles are present in larger amounts, compared to to the larger particles

In order to ascertain that the observed reduction in particles is statistically significant, back calculation of the relation between air sampling size, number, and size of particles measured was done, according to the ISO 14644-1, and a degree of protection of 5.0 for the ≥0.3 µm particles could be demonstrated as being statistically reliable. For the ≥0.5 µm particles, the statistically reliable degree of protection is 3.5 and for the 1.0 µm particles, this value is 2.5 (Fig. 4).

The degree of protection is shown in Fig. 3. It is evident that the large particles, acting as a marker for the larger particles carrying bacteria, are most significantly reduced by use of the UDF setup.

## Discussion

Reduction of endophthalmitis rates for office-based procedures is highly pertinent at this time where frequent and repeated intra-vitreal injections are performed outside an operating room setting.

We observed that the commercially available UDF mobile unit employed in this study enables the reduction of air-borne particles over the instrument table and surgical area during a simulation of an intra-vitreal injection procedure. The degree of protection is at least 5 (*p* < 0.05) over the instrument table and at least 2.64 (*p* < 0.05) in the ocular area for the smallest particles. The background measurements were unaffected. Our results confirm previous findings of other authors [9, 10, 17]. Based on the notion that the particles measured are a surrogate marker for the behavior of particles carrying bacteria (the guiding particles), we conclude that at the instrument table, the degree of protection is significant (*p* < 0.05). However, at the operative site, the degree of protection conveyed also depends on the background particle count. The degree of protection was sufficient, but much more dependent on how many people are in the room and the ventilation of the room itself, a factor which influences the background counts.

There are no universal guidelines for the air quality in treatment rooms where intra-vitreal injections take place. In the literature, there is no evidence that air-borne particles play a role in post-intra-vitreal injection endophthalmitis. A 100-fold decrease of air-borne particles may allow for much safer environment for intra-vitreal injections. Our results show that a degree of protection between 2.39 and 2.55, a reduction of at least 200-fold, can be achieved for the surgical area. We believe that this reduction in particulate matter will convey a good degree of protection for intra-vitreal injections.

It is difficult to extrapolate our findings to the situation in different settings and treatments rooms. For example, in orthopedic surgery, the surface of the implants and the operative areas are many times larger than that of an intra-vitreal injection site, and the procedures leave the patient exposed and vulnerable for much longer times. However, we can make a theoretical calculation of the conditions needed for the UDF to be effective in reducing particle counts to levels acceptable in the most stringent conditions, such as orthopedic surgery. People present in the operating room emit 10 colony-forming units (CFU)/s/person [18]. Previous studies have shown that the outside air carries between 1400 and 2500 CFU/m^3^ [19]. Based on a ventilation rate of 100 m^3^/h without filtration, 2 staff members wearing cuffed mixed-material washed scrubs (emitting 4.2 CFU/s/person) and the patient emitting the same amount of CFU, present in the room with a background concentration between 1854 and 2954 CFU/m^3^, respectively, can be expected [20]. This means that a reduction of 185–295-fold (degree of protection 2.27–2.47) is necessary to realize a concentration of less than 10 CFU/m^3^. Achieving a degree of protection of 2.64 will not require extra measures for air quality (e.g., filtration of the supplied fresh air) in addition to use of the UDF. So, with a degree of protection of at least 2.55, no additional measures are needed at the above stated concentration in open air. In order to prevent a bias, we performed a calculation based on the air sample volume, the number of particles found, and the degree of protection that ensued. This was based on the standards provided by the ISO 14644-1 [16]. The results of our calculations show that for particles of 0.3 microns and larger a degree of protection of 5 is statistically reliable, while for the 0.5 particles this is 3.5, and for particles over 1 micron in diameter a degree of protection of 2.5 suffices. We conclude therefore that in intra-vitreal injections, the instrument table is more than adequately protected with the UDF, and that the adequacy of degree of protection conveyed at the operative site by the UDF is dependent on the background levels of air contamination caused by the ventilation rate of the room, the number of CFU’s in the outdoor/supply air, number of staff, and the used surgical garb.

Our results suggest that the UDF mobile unit may provide an environment for intra-vitreal injections in which the risk of air-borne infection can be markedly reduced. It is clear that the settings would have to be precisely adjusted for each particular site, in order to calculate the actually present degree of protection. In the ambulatory setting of intra-vitreal injections that carry a low, but repetitive risk of a serious complication like endophthalmitis, the use of a UDF unit provides extra safety to the patient, while keeping costs lower than when using a full-fledged operating room.

## Conclusion

In an environment simulating intra-vitreal injection procedures, the unidirectional flow mobile unit has shown a protection factor of up to 5 for the instrument table and a protection factor of 2.64 (*p* < 0.05) to 5 (*p* < 0.05) for the ocular surface. The UDF mobile unit may therefore sufficiently prevent air-borne infections in the setting of intra-vitreal injections.
